# Sleep Duration, Mental Health, and Increased Difficulty Doing Schoolwork Among High School Students During the COVID-19 Pandemic

**DOI:** 10.5888/pcd20.220344

**Published:** 2023-03-16

**Authors:** Sarah A. Sliwa, Anne G. Wheaton, Jingjing Li, Shannon L. Michael

**Affiliations:** 1 Division of Population Health, National Center for Chronic Disease Prevention and Health Promotion, Centers for Disease Control and Prevention, Atlanta, Georgia; 2 Division of Adolescent and School Health, National Center for HIV, Viral Hepatitis, STD, and TB Prevention, Centers for Disease Control and Prevention, Atlanta, Georgia

## Abstract

We estimated the prevalence of short sleep duration (<8 h/average school night) among high school students (grades 9–12) during the COVID-19 pandemic by using data from the Adolescent Behaviors and Experiences Survey (January–June 2021; N = 7,705). An adjusted logistic regression model predicted prevalence ratios for more difficulty doing schoolwork during the pandemic compared with before the pandemic. Most (76.5%) students experienced short sleep duration, and two-thirds perceived more difficulty doing schoolwork. Students who slept less than 7 hours per school night or experienced poor mental health were more likely to report increased difficulty doing schoolwork. Addressing students’ sleep duration could complement efforts to bolster their mental health and learning.

**Summary**

**What is already known on this topic?**

Insufficient sleep among adolescents has been associated with lower academic grades, increased health risk behaviors, and poorer physical and mental health.

**What is added by this report?**

Most high school students were not sleeping enough during the COVID-19 pandemic, which was correlated with poor mental health. Students who experienced short sleep duration were more likely to report greater difficulty doing schoolwork during the pandemic than before the pandemic.

**What are the implications for public health practice?**

Schools can consider including policies and practices known to improve sleep duration within a broader strategy to bolster adolescent mental health and learning.

## Objective

The COVID-19 pandemic has disrupted daily life in many ways, including changes that could either improve or impede sleep duration among adolescents. Periods of remote learning may have provided opportunities for adolescents to sleep late; findings from some small studies suggest adolescents may have shifted to later bedtimes and wake times and slept longer (1–3). Short sleep duration among adolescents is linked to higher risk of injury, worse metabolic and mental health, and difficulty focusing (4). The prevalence of short sleep duration and its association with difficulty doing schoolwork and poor mental health during the COVID-19 pandemic have yet to be explored in a nationally representative sample. Examining this association and estimating the co-occurrence of short sleep duration with poor mental health might provide schools with an additional rationale to adopt policies that lead to improvements in sleep duration within a comprehensive approach to support student academic achievement and mental health.

## Methods

We used data from the Adolescent Behaviors and Experiences Survey (ABES) — a one-time, nationally representative, cross-sectional survey of high school students (grades 9–12) — fielded from January through June 2021 (N = 7,705). The overall ABES response rate during the study period was 18% (school response rate [38%] × student response rate [48%]) (5). Details on ABES are available elsewhere (www.cdc.gov/healthyyouth/data/abes.htm). ABES was reviewed and approved by institutional review boards at the Centers for Disease Control and Prevention and ICF International (5).

Data on sleep duration were derived from the question “On an average school night, how many hours of sleep do you get?” (Table 1). To align with American Academy of Sleep Medicine recommendations (8–10 h for ages 13–18 y) (4), we restricted analyses to participants who reported their age as 13 years or older. We combined the response options for 8, 9, and 10 or more hours into a single category (≥8 h), which resulted in 5 categories (≤4, 5, 6, 7, or ≥8 h). We defined short sleep duration as sleeping ≤4, 5, 6, or 7 hours and ≥8 hours as meeting sleep recommendations. Data on the primary outcome was derived from the question “Do you agree or disagree that doing your schoolwork was more difficult during the COVID-19 pandemic than before the pandemic started?” with “strongly agree” and “agree” coded as experiencing “more difficulty doing schoolwork” vs “not sure,” “disagree,” or “strongly disagree.”

**Table 1:** Analytic Variables Derived from the Adolescent Behaviors and Experiences Survey, United States, January–June 2021

Type	Variable name	Survey item	Variable construction
Primary outcome	More difficulty doing schoolwork during pandemic	Do you agree or disagree that doing your schoolwork was more difficult during the COVID-19 pandemic than before the pandemic started?	More difficulty doing schoolwork: strongly agree or agree vs not sure or disagree or strongly disagree
Primary predictor	Sleep duration	On an average school night, how many hours of sleep do you get?	• ≤4 hours • 5 hours • 6 hours • 7 hours • Meets recommended ≥8 hours (8 hours, 9 hours, or ≥10 hours)
Covariate	Self-reported hunger	During the COVID-19 pandemic, how often did you go hungry because there was not enough food in your home?	Experienced self-reported hunger: always or most of the time or sometimes vs never or rarely
	Poor mental health	During the COVID-19 pandemic, how often was your mental health not good? (Poor mental health includes stress, anxiety, and depression.)	Experienced poor mental health: always or most of the time vs sometimes or rarely or never
	High screen time	On an average school day, how many hours do you spend in front of a TV, computer, smart phone, or other electronic device watching shows or videos, playing games, accessing the Internet, or using social media (also called “screen time”)? (Do not count time spent doing schoolwork.)	• High level of screen time (≥5 hours/day)^a^ • <5 hours/day (<1 hour/day, 1 hour/day, 2 hours/day, 3 hours/day, or 4 hours/day)
	Sex	What is your sex?	• Female • Male
	Grade	In what grade are you?	• 9th grade • 10th grade • 11th grade • 12th grade • Ungraded or other grade
	Race and ethnicity	Developed from Q4. Are you Hispanic or Latino? Q5. What is your race? (Select 1 or more responses.)	• Hispanic • Non-Hispanic Black • Non-Hispanic White • Non-Hispanic American Indian or Alaska Native; Asian, Native Hawaiian or Other Pacific Islander, and multiracial

a Rather than setting a time limit, the American Academy of Pediatrics recommends placing consistent limits on the time spent using media and the types of media for adolescents and ensuring that media does not take the place of adequate sleep, physical activity, and other behaviors essential to health. From previous studies, we defined high level of screen time by using the largest response category: ≥5 hours per day.

Bivariate analyses (χ^2^ tests, Pearson correlation) and univariate logistic regression models (Wald *F* test) assessed the associations between difficulty doing schoolwork, short sleep duration, and selected covariates (poor mental health, high level of screen time [≥5 h/d], and self-reported sex, race and ethnicity, grade, and hunger). An adjusted logistic regression model predicted prevalence ratios (PRs) for experiencing more difficulty doing schoolwork, including the covariates. We tested whether poor mental health modified the association between sleep duration and more difficulty doing schoolwork (interaction term: sleep duration × poor mental health). Analyses were conducted in SAS-callable SUDAAN version 11.0.3 (RTI International) and used sample weights to account for complex sampling and nonresponse. Statistical significance was set at *P* < .05.

## Results

The sample was evenly distributed across sex and grade and racially and ethnically diverse; no racial or ethnic group comprised a majority (Table 2). Most high school students (76.5%) experienced short sleep duration, and 66.6% reported more difficulty doing schoolwork during the COVID-19 pandemic than before the pandemic.

**Table 2:** Demographic Characteristics, Behaviors, and Experiences of Participants in Adolescent Behaviors and Experiences Survey (ABES) (N = 7,705), United States, January–June 2021^a^

Characteristic	No. of participants	% (95% CI)
**Sex**		
Female	7,677	50.4 (46.9–53.9)
Male		49.6 (46.1–53.1)
**Race and ethnicity**		
Hispanic or Latino	7,632	25.4 (20.1–31.6)
Non-Hispanic AIAN, Asian, NHPI, and multiracial		11.9 (8.3–16.9)
Non-Hispanic Black		12.9 (9.3–17.5)
Non-Hispanic White		49.8 (41.5–58.1)
**Grade**		
9	7,682	26.7 (24.0–29.6)
10		25.5 (23.1–28.0)
11		24.3 (22.3–26.4)
12		23.5 (21.2–26.1)
**Perceived more difficulty doing schoolwork than before the pandemic started^b^**	7,171	66.6 (64.5–68.6)
**Sleep duration on average school night, h**		
≤4	7,074	11.6 (9.8–13.7)
5		13.9 (12.5–15.4)
6		24.8 (23.3–26.3)
7		26.2 (24.8–27.6)
≥8 (met sleep recommendation)		23.5 (21.6–25.7)
**Experienced self-reported hunger during the COVID-19 pandemic^c^**	7,181	8.4 (7.2–9.8)
**Experienced poor mental health during the COVID-19 pandemic^d^**	7,207	37.1 (34.6–39.6)
**High level of screen time (≥5 h on average school day)^e^**	7,210	48.6 (46.1–51.2)

Abbreviations: AIAN, American Indian or Alaska Native; NHPI, Native Hawaiian or Other Pacific Islander.

a Ns are unweighted, percentages are weighted.

b Respondents answered “agree” or “strongly agree.”

c Respondents answered “always,” “most of the time,” or “sometimes.”

d Respondents answered “always” or “most of the time.”

e Hours of screen time encompass time spent “in front of a TV, computer, smart phone, or other electronic device watching shows or videos, playing games, accessing the Internet, or using social media (also called *screen time*),” not including time spent doing schoolwork.

Overall, 37.1% reported poor mental health during the pandemic, which correlated with short sleep duration (Pearson correlation *r* = 0.22, *P* < .001; χ^2^_4_ *=* 347.48, *P* < .001). Among students who met sleep recommendations, 25.2% reported poor mental health. About half of students who slept 5 (49.1%) or 4 hours or less (55.9%) reported poor mental health (*P* < .001).

The unadjusted models confirmed the hypothesized association between short sleep duration and greater difficulty doing schoolwork, which remained robust after adjusting for covariates (Table 3). Students who slept less than 7 hours during an average school night had a significantly greater prevalence of experiencing more difficulty doing schoolwork during the COVID-19 pandemic compared with students who met sleep duration recommendations (6 h sleep: PR = 1.17 [95% CI, 1.08–1.27]; 5 h sleep: PR = 1.18 [95% CI, 1.09–1.28]; ≤4 h sleep: PR = 1.20 [95% CI, 1.08–1.33] vs ≥8 h sleep).

**Table 3:** Association Between High School Students’ Sleep Duration and Greater Difficulty Doing Schoolwork During the COVID-19 Pandemic — Adolescent Behaviors and Experiences Survey, United States, January–June 2021

Variable	Unadjusted^a^^,^ b prevalence ratio (95% CI)	Adjusted^b^^,^ c prevalence ratio (95% CI)
**Sex**		
Female	1.08 (1.03–1.13)^d^	1.03 (0.98,1.09)
Male	Reference	Reference
**Race and ethnicity**		
Hispanic or Latino	1.06 (1.00–1.13)^d^	1.08 (1.01–1.15)^d^
Non-Hispanic AIAN, Asian, NHPI, and multiracial	0.99 (0.91–1.09)	0.98 (0.89,1.08)
Non-Hispanic Black	1.03 (0.96–1.11)	1.03 (0.96–1.1)
Non-Hispanic White	Reference	Reference
**Grade**		
9	Reference	Reference
10	1.01 (0.94–1.07)	1.00 (0.94–1.07)
11	0.98 (0.92–1.05)	0.97 (0.90–1.04)
12	1.00 (0.92–1.08)	0.98 (0.91–1.05)
**Sleep duration on average school night, h**		
≤4	1.26 (1.14–1.38)^d^	1.20 (1.08–1.33)^d^
5	1.21 (1.12–1.31)^d^	1.18 (1.09–1.28)^d^
6	1.19 (1.10–1.28)^d^	1.17 (1.08–1.27)^d^
7	1.08 (1.00–1.18)	1.08 (1.00–1.18)
≥8 (met sleep recommendation)	Reference	Reference
**Self-reported hunger during the COVID-19 pandemic**		
Always/most of the time/ sometimes	1.11 (1.04–1.19)^d^	1.07 (0.99–1.16)
Rarely/never	Reference	Reference
**Poor mental health during COVID-19 pandemic**		
Always/most of the time	1.21 (1.14–1.27)^d^	1.17 (1.10–1.25)^d^
Sometimes/rarely/never	Reference	Reference
**High level of screen time on average school day**		
Yes (≥5 h)	0.99 (0.95–1.04)	0.95 (0.91–1.00^e^)
No (<5 h)	Reference	Reference

Abbreviations: AIAN, American Indian or Alaska Native; NHPI, Native Hawaiian or Other Pacific Islander.

a Models present prevalence ratios calculated from predicted marginals from univariate logistic regressions. Each row presents the value from a regression model specific to that variable.

b Sample size fluctuates for univariate models and is smaller for the fully adjusted model (N = 6,903) because of missing values.

c Single model adjusted for self-reported hunger, poor mental health, screen time, sex, race or ethnicity, and grade.

d *P* < .05 as indicated by Wald *F* test in logistic regression models.

e Nonrounded value is 0.997, *P* = .03.

Students who experienced poor mental health had 17% higher prevalence of more difficulty doing schoolwork compared with students who did not report poor mental health (PR = 1.17; 95% CI, 1.10–1.25). Poor mental health did not moderate the association between short sleep duration and more difficulty doing schoolwork. Students with 5 or more hours of screen time were slightly less likely to report more difficulty doing schoolwork than students who spent less than 5 hours per day using screens (PR = 0.95; 95% CI, 0.91–0.997). Self-reported sex, grade, and hunger were not associated with more difficulty doing schoolwork in the adjusted model. Hispanic or Latino students were more likely to report more difficulty doing schoolwork than White students.

## Discussion

Before the COVID-19 pandemic, short sleep duration was becoming more prevalent among US high school students (74.6% [73.1%–76.0%] in 2017 and 77.9% [76.3%–79.4%] in 2019, *P* < .001) (6). We found that short sleep duration remained widespread during the COVID-19 pandemic, affecting roughly three-quarters of students. Students who slept less than 7 hours during an average school night were more likely to report greater difficulty doing schoolwork during the pandemic compared with before the pandemic, as were those who experienced poor mental health. This study contributes to the literature by highlighting the co-occurrence of short sleep duration and poor mental health during the COVID-19 pandemic. Teachers have identified behavioral and mental health challenges among the leading barriers to addressing learning gaps during the 2022–2023 school year (7). Policies known to improve sleep duration among students, including later school start times and family practices, such as good sleep habits and parent-set bedtimes, might help support both learning and mental health (8,9).

Previously noted limitations to ABES include the low response rate and the inability to draw causal inferences about the impact of the COVID-19 pandemic (5). We note additional limitations. The recall period differed across some of the items: the sleep and screen time questions reference the average school day or night, whereas items about mental health and greater difficulty doing schoolwork reference “during the COVID-19 pandemic.” We do not know how students interpretated “average school day.” Rapid survey development during the COVID-19 pandemic precluded item validation and testing. Previous univariate models confirmed small significant associations in the expected direction between short sleep duration, self-reported hunger (10), and poor mental health (4), which suggests concurrent validity. We cannot account for some potential confounders, such as socioeconomic status and school instruction modality (eg, remote, hybrid, in-person). The latter may have influenced wake times and sleep duration, even if school start times were unchanged. Instruction modality may also have influenced self-reported screen time; the question excludes time doing schoolwork but does not specify whether to count time using screens to *attend* school. This may help explain the counterintuitive finding that a high level of screen time was inversely associated with greater difficulty doing schoolwork. ABES addressed a single dimension of sleep; we could not assess sleep quality, sleep schedules, or sleep disorders.

Nevertheless, our findings show that most students were not sleeping enough and that many students concurrently experienced poor mental health and insufficient sleep. Schools can consider addressing sleep duration within a broader strategy to bolster adolescent mental health and learning, including addressing protective factors (11,12).
